# Progress in State Medicine

**Published:** 1903-07-04

**Authors:** 


### Progress in State Medicine.

Sewage.—Attention[1] is drawn to the fact that, in regard to drainage, Nottingham is altogether behind the age, with the result that typhoid fever is endemic and periodically assumes the epidemic form, the mortality, according to the Registrar-General's returns, being 0·35 per 1,000 of the population—higher than that of any other town. This is attributed to the "pail system" in vogue in the city. In 1897 there were still 40,225 pail-closets in Nottingham, and these were for the most part made of wood. There were only 7,200 water-closets and 400 privies. In the 10 years 1887–1896 the maximum number of cases of typhoid occurred in 1887, when one case of typhoid was recorded for every 97 houses that had pail-closets. The minimum was in 1892, when there was one case for every 218 houses with pail-closets. In houses having water-closets the maximum number of cases of typhoid fever was one case per 280 houses in 1893, and the minimum one case per 795 houses in 1892. Summarising these figures for the 10 years shows that there was one case of typhoid fever for every 120 houses that had pail-closets, one case for every 37 houses with privy-middens, and one case for every 558 houses with water-closets. Wherever introduced water-closets have assured relative immunity from typhoid fever. In 1901 there was one case of typhoid in every 84 houses with pail closets, one in every 12 houses with midden privies, and one in every 255 houses with water-closets. Difficulty is sometimes experienced in getting rid of the night-soil, as much as 6,000 tons having been accumulated at a depôt near to a district of Nottingham, and it has been noticed that during the prevalence of typhoid the wind for the greater part of the time blew over this depôt towards the district that suffered most from the fever. Clowes,[2] in stating the results of his experiments of the bacterial treatment of crude London sewage, describes how the sewage, straight from the main sewer, is first screened from its coarser matters, and then pumped continuously into a settling tank, the rate of supply being so adjusted to the capacity of the tank that the sewage remains about six hours in the tank before it flows away through an elbow pipe beneath the surface of the liquid into the coke-beds. In this tank practically the whole of the suspended or floating particles of the sewage settle as sludge, and are allowed to remain undisturbed. It is found that a considerable proportion of this sludge disappears by bacterial action. The sewage, as it flows from the settling-tank, is less pure than the effluent which is at present discharged into the river at Crossness, and is quite unsuitable for direct discharge into the stream. But by the subsequent coke-bed treatment it is rendered sufficiently pure to support the life of fish, and to ensure it against undergoing, even in summer time, any offensive change. In the experimental treatment the settled sewage is therefore allowed to flow into a coke-bed, which is a tank filled with suitable coke fragments to a depth of 6 feet. As soon as the bed is filled to the surface of the coke the supply is stopped, and the sewage liquid is allowed to remain in contact with the coke for two hours. It is then drained off from the bottom of the bed and constitutes the "bacterial effluent." After the liquid has flowed away, the coke-bed remains empty for two hours, and is then refilled with a fresh supply of the settled sewage, which is treated in precisely the same way as before. His conclusions from these experiments are that (i.) by suitable, continuous, undisturbed sedimentation the raw sewage is deprived of matter which would choke the coke-beds, and the sludge which settles out is reduced in amount by bacterial action to a very considerable extent; (ii.) that the coke beds, after they have developed their full purifying power by use, have an average sewage capacity of about 30 per cent. of the whole space which has been filled with coke; (iii.) that the sewage capacity of the coke-bed, when the bed is fed with settled sewage, fluctuates slightly, but undergoes no permanent reduction, the bed does not choke, and its purifying power undergoes steady improvement for some time; (iv.) that coke of suitable quality does not disintegrate during use; (v.) that the "bacterial effluent" of settled sewage from the coke-beds does not undergo offensive putrefaction at all, even in summer heat, and can never become offensive. It also satisfactorily supports the respiration of fish. Scoble,[3] speaking of rural drainage, states that the two most pertinent questions to be considered are, (1) at what point in the growth or development of a parish does a drainage system become necessary, and (2) granted that the parish has developed sufficiently for a drainage system, what kind shall be adopted—drainage by sewer or drainage by vans (known as the Bexley system)? With regard to the latter, most scavengering schemes are doomed to failure by reason of the cost of collection and transport. If ordinary sewerage is determined on, he is convinced, on the grounds of efficiency and economy, that the general adoption of the bacterial treatment of sewage is a mere matter of time. The initial outlay for chemical precipitation works and bacterial installations does not present any great divergence. The value of the effluent is an aspect of the sewage problem which has been neglected. It has been calculated that some 90 per cent. of nitrogen value of sewage can be converted into nitrate by biological treatment; it would seem worth while to take some steps to utilise, as far as possible, this manure. He advocates the establishment of watershed authorities, having as their duties the supervision of water supply, sewage disposal, and dust destruction. The chief advantages of the anærobic treatment of sewage are—(1) that the effluent is rendered practically uniform, therefore lending itself more readily to further purification; (2) cellulose, *i.e.*, wood, paper, vegetable tissue, undergoes decomposition more quickly than in the interstices of a bacterial bed; (3) that no fall is required, because the liquid leaves the tank at the same level as the sewer invert. The provision of two successive bacterial beds to treat septic tank effluent seemed, from experiments, to be scientifically correct, as it will allow of the production of nitrites in the first and nitrates in the second bed. Before the solution of the important question of rural drainage can be attained, the onerous conditions insisted upon by the Local Government Board must be altered. This body appears still to cling to land for final treatment and purification, thus failing to grasp the fundamental principle that bacterial treatment is essentially only an improved form of land treatment, wholly under control and efficient in all states of weather. It is to be hoped that in the final report of the Royal Commission on Sewage, shortly to be issued, substantial modifications of its present regulations will be permitted, or, rather, insisted upon, otherwise the Local Government Board will act as an incubus instead of being a help to local bodies, by causing an urgent matter to be postponed to as late a day as possible, and by saddling owners of property with an altogether excessive burden. Statistics dealing[4] with the cost of maintenance of the sewage disposal works at Sutton (Surrey), which are mainly bacterial, show at what small cost a properly-managed farm can be maintained whilst purifying the sewage in a thoroughly effectual manner. The quantity of sewage treated for the year ending March 31st last was 194,695,000 gallons, and of this amount one-third has to be pumped to the high-level when it reaches the farm. The net cost of treating this quantity of sewage, including pumping, was £775, equivalent to a cost of £4 1s. 7½d. per million gallons, or 11·07d. per head of population, whilst the charge on the rates amounted to 1¾d. only, and during this period an uniformly good effluent had been maintained, which was discharged into a brook of very small volume, under the jurisdiction of the Thames Conservancy. The sewage farm has an area of 25¼ acres, which consists of stiff clay and ill-adapted for sewage purification, but only 13¾ acres are available for irrigation. The surface of the land was converted from an impervious clay to a friable soil by the addition of several thousand loads of domestic ashes which were well ploughed in. Of the 13¾ acres under cultivation 8 acres were devoted in 1901–2, and 6¾ acres in 1902–3, to the growing of peppermint, which plant has been found one of the most profitable crops which could be grown on a sewage farm. A revenue of £235 was obtained in 1901–2 from this source, and of £178 15s. in 1902–3. The bacteria beds are 12 in number, six being coarse-grain and six fine-grain beds, the former being composed in part of burnt-clay ballast and furnace clinkers, and the latter of coke breeze and fine burnt clay ballast. The use of the last-named material is not being extended as its efficiency is not maintained to that extent which justifies its continuance on the grounds of economy, as compared with a less pulverisable material, such as furnace clinkers. Gaskell,[5] in summarising the work of the West Riding Rivers Board during the past 10 years, points out that the practice of throwing solid refuse into the streams—so universal in the past—had been virtually stopped with the exception of the sludging of mill dams. In 1893 in the five county boroughs there were eight sewage works. To-day, in the six county boroughs there were 17. There were 13 non-county boroughs with 13 sewage works in 1893; now there were 14 non-county boroughs with 28 works; there were 124 urban districts with 57 works, now there were 117 districts with 117 works. In 1893, 29 rural districts had 48 works as compared with 105 works at the present day. The total volume of sewage daily poured into the streams by the six county boroughs of the West Riding was 60,000,000 gallons, and as long as the present pollution continued no satisfactory result could be attained. The longer the delay in dealing with the evil, the greater the cost would be. In 1893 there were six non-county boroughs which had no sewage works, to-day there was only one, Todmorden, which was at present carrying out a scheme. In 1893 81 urban districts had no sewage works, now there were only 39 in that condition, and of these 16 had schemes in progress or sanctioned by the Local Government Board, and 11 had schemes in preparation. Much less solid refuse was discharged from manufacturing processes than formerly, the owners finding it more profitable to erect their own purification works.

[1] Lancet, Nov. 29, 1902. [2] Sanit. Rec., Dec. 4, 1902. [3] Ibid., Jan. 22, 1903. [4] Ibid. [5] Public Health Engineer, April 25, 1903.

